# Clinicopathological features and prognostic value of SOX11 in childhood acute lymphoblastic leukemia

**DOI:** 10.1038/s41598-020-58970-z

**Published:** 2020-02-06

**Authors:** Toni Grönroos, Artturi Mäkinen, Saara Laukkanen, Juha Mehtonen, Atte Nikkilä, Laura Oksa, Samuli Rounioja, Yanara Marincevic-Zuniga, Jessica Nordlund, Virva Pohjolainen, Timo Paavonen, Merja Heinäniemi, Olli Lohi

**Affiliations:** 1 Tampere Center for Child Health Research, Faculty of Medicine and Health Technology, Tampere University, Tampere, Finland; 2 Fimlab Laboratories, Department of Pathology, Tampere University Hospital, Tampere, Finland; 3 Institute of Biomedicine, School of Medicine, University of Eastern Finland, Kuopio, Finland; 4 Fimlab Laboratories, Department of Hematology, Tampere University Hospital, Tampere, Finland; 5 Department of Medical Sciences, Molecular Medicine and Science for Life Laboratory, Uppsala University Uppsala, Sweden; 6 Department of Pathology, Faculty of Medicine and Health Technology, Tampere University, Tampere, Finland; 7 Department of Pediatrics, Tampere University Hospital, Tampere, Finland

**Keywords:** Haematological cancer, Acute lymphocytic leukaemia

## Abstract

Acute lymphoblastic leukemia is marked by aberrant transcriptional features that alter cell differentiation, self-renewal, and proliferative features. We sought to identify the transcription factors exhibiting altered and subtype-specific expression patterns in B-ALL and report here that SOX11, a developmental and neuronal transcription factor, is aberrantly expressed in the ETV6-RUNX1 and TCF3-PBX1 subtypes of acute B-cell leukemias. We show that a high expression of *SOX11* leads to alterations of gene expression that are typically associated with cell adhesion, migration, and differentiation. A high expression is associated with DNA hypomethylation at the *SOX11* locus and a favorable outcome. The results indicate that SOX11 expression marks a group of patients with good outcomes and thereby prompts further study of its use as a biomarker.

## Introduction

SOX11 is a transcription factor (TF) encoded by the *SOX11* gene located in chromosome 2p25^1^. It is a member of the *SoxC* (sex-determining region Y-related HMG box) group of genes and consists of two functional domains—the N-terminal DNA-binding and the C-terminal transactivation domains^2,3^. Other SoxC family members include SOX4 and SOX12. Of these, SOX4 is a crucial TF in B lymphopoiesis and is expressed in the B- and T-cell lineages^2,4,5^. SOX11 is normally expressed in the developing central nervous system of the embryo, in keratinocytes, and in some other epithelial tissues^1,6–8^. It is also expressed in ovarian and breast cancer, in which both tumor suppressor and oncogenic functions have been suggested^9,10^. A knockout mouse model revealed the vital role of *SOX11* during embryonic development, as *SOX11*-deficient mice died after birth likely from multiple heart defects, asplenia, and organ hypoplasia in the lungs, stomach, and pancreas^11^. *SOX11* deletions and mutations are associated with neurodevelopmental disorders^12^.

Previous studies have shown that SOX11 mRNA and nuclear protein expression is a specific marker for conventional but not indolent mantle cell lymphoma (MCL)^8,13–15^. In MCL, SOX11 has been associated with either increased or reduced cell proliferation^16–23^ and either good or bad prognosis^24–27^. In a cohort of 50 adult acute myeloid leukemia (AML) patients, a high *SOX11* expression was associated with FLT/ITD and NPM1 mutations and a shortened disease-free survival^28^.

There is other evidence linking SOX11 with B-lineage malignancies. Dictor *et al*. (2009) reported that the nuclear expression of SOX11 was found in eight cases of B-lymphoblastic neoplasias^29^. Another study reported the strong nuclear expression of SOX11 in a single B-cell and five T-cell lymphoblastic lymphoma/leukemias^13^. Vegliante *et al*. (2011) demonstrated the increased expression of *SOX11* mRNA in ETV6-RUNX1 (E/R) and TCF3*-*rearranged B-cell precursor acute lymphoblastic leukemia (BCP-ALL)^30^, whereas Nordlund *et al*. (2012) and Busche *et al*. (2013) observed the prominent expression of *SOX11* in the E/R subtype of ALL^31,32^.

We investigated the expression of SOX11 across lymphoid malignancies, focusing on B-lymphoblastic leukemias. The function of SOX11 in leukemias and its clinical significance as a biomarker were further explored.

## Materials and Methods

### Microarray datasets

We used three independent datasets to study *SOX11* expression—a combined microarray dataset (“Hemap”) retrieved from Gene Expression Omnibus (GEO)^33,34^, the GEO series GSE47051^35^, and the publicly available BCP-ALL data from the recent PanALL study^36^. The sample sizes for each dataset are shown in Supplementary Table 1.

### Cell lines, cell culture, and drug treatments

NALM-6, REH, 697, RCH-ACV, KOPN-8, KASUMI-2, JURKAT, MOLT-16, P12-ICHIKAWA, HPB-ALL, and CCRF-CEM were cultured in RPMI Medium 1640 (Gibco, Thermo Fisher Scientific, Waltham, MA, USA) with 2 mM L-glut, 100 U penicillin, 100 µg/ml streptomycin with 10% FBS (Gibco), and MOLT-4, PEER, and MHH-CALL3 with 20% FBS (Gibco) at 37 °C in 5% CO_2_. An inducible *E/R* fusion in the NALM-6 cell line and a knockdown of *E/R* by a short hairpin RNA (shRNA) in the REH cell line have been previously described^37^. *E/R* expression was induced with 500 ng/ml doxycycline (Clontech). *E/R* expression changes were confirmed with RT-qPCR, with fusion gene-specific primers (Table S2). Mycoplasma tests were done regularly for the cell lines, and Eurofins Genomics (Ebersberg, Germany) services were used to authenticate the cell lines by STR genotyping. All cell lines used in this study were purchased from the Leibniz Institute DSMZ-German Collection of Microorganisms and Cell Cultures (Braunschweig, Germany).

For the methyltransferase inhibition experiments, the cultured cells were treated for 72 h with decitabine, 5-Aza-2′-deoxycytidine solved in DMSO (A3656, Sigma-Aldrich, St. Louis, MO, USA) at 0, 0.1, and 1 µM concentrations. The media were changed at a 24 h interval to compensate for decitabine instability under cell culture conditions. After the treatment cells were collected, RNA was extracted for RT-qPCR analyses. Corticosteroid and chemotherapy treatments were conducted with the indicated concentration ranges, and cell viabilities were measured after either 72 (697 and RCH-ACV) or 96 h (REH). The corticosteroids included prednisolone (P6004, Sigma-Aldrich) and dexamethasone (D8893, Sigma-Aldrich), and the chemotherapy agents used were asparaginase (A3809, Sigma-Aldrich) and vincristine (V8879, Sigma-Aldrich). The applied concentrations for each cell line are indicated in Table S3.

### Quantitative real-time PCR

Total RNA was extracted using the PureLink™ RNA Mini Kit, and the On-Column PureLink® DNase Treatment Protocol was used for DNA removal (Ambion® by Life Technologies and Invitrogen, Thermo Fisher Scientific, Waltham, MA, USA); 100–500 ng of the extracted RNA was used as a starting material for cDNA synthesis, which was performed with iScript (Bio-Rad, Hercules, CA, USA). RT-qPCR reactions were conducted according to the manufacturer’s instructions with SsoFast EvaGreen® Supermix (Bio-Rad). The following program was performed with the Bio-Rad CFX96^TM^ Real-Time System (Bio-Rad): initial denaturation at 96 °C for 30 s, 39 cycles of denaturation at 96 °C for 2 s, annealing at 60 °C for 5 s, and plate read. Independent experiments performed in triplicate were used as the starting material for the RT-qPCR measurements, and the relative 2^−ΔΔC^_T_ method was used for quantification^38^. The primer sequences used in RT-qPCR are listed in Table S2.

### Western blot

Protein extraction was performed using M-PER reagent (Thermo Fisher Scientific) according to the manufacturer’s instructions to lyse the cells, and protein concentrations were measured with *DC* Protein Assay (Bio-Rad); 15–20 µg of a protein sample was loaded into the precast 10% Mini-PROTEAN® TGX Stain-Free^TM^ Gels (Bio-Rad). After the electrophoresis run, Trans-Blot® Turbo^TM^ Pack (Bio-Rad) was used to transfer the proteins from the gel to the nitrocellulose membrane. Transfer was done with the Trans-Blot® Turbo^TM^ Transfer system according to the manufacturer’s instructions (Bio-Rad). A prestained protein ladder, PageRuler Plus (#26619, Thermo Fischer Scientific), was used as a protein size marker. We utilized antibodies against SOX11 (1:1,000) (HPA000536, Sigma-Aldrich, Lot # BB107024) and Histone H3 (1:75,000) (ab4729, Abcam, Cambridge, UK, Lot # GR167613-1). Horseradish peroxidase conjugated anti-rabbit (1:2,000) (P0217, Lot # 00069121) was used as a secondary antibody (Agilent Technologies, Santa Clara, CA, USA). Chemiluminescence reaction by Amersham ECL reagent was detected with ChemiDoc^TM^ XRS+using Image Lab^TM^ Software (Bio-Rad).

### Gene silencing with nucleofection

Knockdowns for suspension cells were performed using 4D-Nucleofector™ (Lonza, Basel, Switzerland) for transfections. *SOX11* knockdown was done using gene sequence-specific small interfering RNA (siRNA) (Sigma-Aldrich), and a non-specific siRNA was used as a control (Table S4). Before nucleofection, 20 µM stock solutions were diluted in Resuspension Buffer (SR30005, OriGene, Rockville, MD, USA) so that the final concentration per reaction was 300 nM. One million cells were used for each nucleofection reaction. Nucleofection reactions were conducted in proper solutions and programs according to the manufacturer’s instructions in single nucleocuvettes (Table S5). Then, the cells were transferred to 12-well plates with prewarmed fresh media. The transfected cells were used in cell viability assays and RNA sequencing (RNA-seq). Western blot and RT-qPCR were used to assess knockdown levels.

### RNA sequencing of cell lines

*SOX11*-specific and control siRNAs were used in the nucleofection for the 697, RCH-ACV, and REH cell lines to create *SOX11* knockdown and control samples. After 48 h of nucleofection, three million cells per sample were collected for the total RNA extraction. RNA extraction was performed with the PureLink™ RNA Mini Kit, and the On-Column PureLink® DNase Treatment Protocol was used to avoid contamination by genomic DNA (Ambion® by Life Technologies and Invitrogen). Protein samples were also collected, and the *SOX11* knockdown level was verified by both RT-qPCR and Western blotting. Three independent biological replicates were collected for each transfected cell line, and each sample had 25 ng of RNA in 40 µl. Library preparation and RNA-seq (GSE123943) were performed in the Finnish Functional Genomics Centre (Turku, Finland). See more details in Supplementary Information.

The quality of the raw sequencing reads was ensured with FastQC (v0.10.1). Based on the FastQC results, reads were trimmed and their quality was filtered using the FASTX Toolkit (0.0.14). The reads were mapped to the human reference genome version hg19 using STAR aligner software (2.5.3a modified); reads aligning to more than two locations were discarded^39^. The alignment file was turned into tag directories, and read counts were calculated using the HOMER tool kit (v4.8).

Differential gene expression was analyzed using the quasi-likelihood F-test from edgeR, an R package^40^. Differentially expressed genes from all cell lines were filtered using the adjusted p-value < 0.05 (Benjamini–Hochberg method) as a cut-off, and the resulting gene lists from three different cell lines were compared by drawing a Venn diagram with an interactive Venn online tool (http://www.interactivenn.net/). Heatmaps, presenting all biological replicates, were drawn by using the z-scores of reads per kilobase of transcript, the per million mapped reads (RPKM) normalized count matrix, and the ComplexHeatmap R-package^41^. Gene set enrichment was analyzed by Gene Set Enrichment Analysis (GSEA) 3.0 software using logFC ranked lists of genes from differential gene expression analysis, and the results presented had an adjusted p-value < 0.02 (Benjamini–Hochberg method)^42,43^. Gene ontology (GO) term enrichment was studied with two approaches—with GSEA software using f-statistics ranked lists of genes and with the GOrilla online tool using unranked target and background lists^44,45^. The target lists were created with a threshold of adjusted p-value < 0.05 and logFC >0.5 or <−0.5.

### RNA sequencing and methylation analysis of the patient samples

Previous transcriptome sequencing of pediatric ALL cohort by Marincevic-Zuniga *et al*.^46^ included 116 BCP-ALL cases, of which 115 cases were used to assess the SOX11 mRNA expression level in this study. DNA methylation data (GSE49031) were available for 112 of the 115 BCP-ALL cases^35^. See more details in Supplementary Information.

### Cell viability and proliferation assays

Fresh media were replaced on the transfected cells after 24 h of transfection. For the cell viability assay, the cells were counted, and 10,000 cells per well were used in a 96-well plate. The cells were allowed to grow up to 72 (697 and RCH-ACV) and 120 h (REH), and cell viability was measured every 24 h with 10 µl of Alamar Blue reagent per well with a 2 h incubation before fluorescence measurement with excitation of 560 nm and emission of 590 nm using the Tecan fluorometer Infinite 200 (Tecan, Männedorf, Switzerland). For each time point, we used four technical replicates per sample to calculate the mean.

Cell proliferation assays were performed by counting the cells every 24 h after the transfection up to 96 (697 and RCH-ACV) and 120 h (REH).

### Clinical data and the patient samples

The data on pediatric ALL cases below 18 years of age and treated in the Tampere University Hospital were retrieved from the clinical registry from years 1990 to 2017. Essential clinical information, such as age, leukocyte count, gender, relapse, death, central nervous system leukemia, immunophenotype based on flow cytometry, and clinical genetic information, was collected. The study obtained permission from the local ethical committee, and informed consent was sought, as needed (Pirkanmaa Hospital District Ethical Committee, R16054 and R13109). The use of old biopsy samples was approved by the National Supervisory Authority for Welfare and Heath (Valvira), and the samples were handled in accordance with relevant guidelines and regulations.

A total of 126 representative diagnostic formalin-fixed and paraffin-embedded (FFPE) decalcified bone marrow trephine biopsy samples were collected from the pathology department archives on the basis of the primary sample reports. For a proportion of cases, remission and relapse samples were also retrieved. Plastic-embedded and inadequate samples were excluded. The cases were classified based on the WHO 2017 Classification of Tumors and Haematopoietic and Lymphoid Tissues^47^.

### Immunohistochemistry

Four micron-thick whole tissue sections were used for immunohistochemistry. All cases were stained with anti-SOX11 antibody (clone MRQ-58, Cell Marque, Sigma-Aldrich, Lot # 1331005 A and 1430213 C) using Ventana Benchmark Classic at a dilution of 1:50. FFPE human MCL was used as a positive control material, and remission bone marrow samples were used as negative controls.

Staining intensity was graded, and cases with less than 20% of positivity of leukemic blast cell nuclei were interpreted as negative. Cases with immunohistochemical positivity ranging from 20% to 50% in the blast cell nuclei were graded positive, and cases with immunohistochemical nuclear positivity of over 50% were considered strongly positive. SOX11 expression was independently analyzed with a light microscope by two experienced pathologists without knowledge of the clinical data. Cases with discrepant scores were further analyzed by a third pathologist.

### Fluorescence *in situ* hybridization

For cases lacking the genetic subtype information, fluorescence *in situ* hybridization analysis was performed on either the bone marrow aspiration samples or the FFPE samples. The following probes were used: Metasystems E2A Break Apart Probe 19p13 (Lot # 18216), Metasystems XL MLL plus Break Apart Probe 11q23 (Lot # 18451), Metasystems XL BCR/ABL1 plus (Lot # 19082), and Metasystems XL t(12;21) (Lot # 19133).

### Flow cytometry analysis

For flow cytometry analysis, the cells were permeabilized using Fix&Perm reagent according to the manufacturer’s instructions (GAS003, Invitrogen). The cells were then stained with Alexa Fluor 647® conjugated rabbit anti-human SOX11 antibody [EPR8191 (2)] (ab198540, Abcam), while CD3-APC antibody (345767) (Becton Dickinson, Franklin Lakes, NJ, USA) served as a negative control. The data were acquired with the Beckman Coulter Navios cytometer (Beckman Coulter, Brea, CA, USA) using Red laser (638 nm) and 660/20 bandpass filter. Data analysis was conducted using Kaluza software (Beckman Coulter).

### Statistical analysis

IBM SPSS Statistics (v. 22) and R (v. 3.40) were used for the statistical analysis. Kaplan–Meier survival analysis was performed, and Log-rank test was used, with a p-value < 0.05 considered statistically significant. Event-free survival (EFS) was analyzed using death, relapse, resistant disease (blast count >25% at the end of induction), and secondary malignancy as events. Cox proportional hazards models were fitted to estimate the effects of potential risk factors on survival. Chi-squared test was performed on SOX11 expression and clinicopathological prognostic variables. Kruskal–Wallis H and Mann–Whitney U tests were used to evaluate the differential expression of *SOX11* in distinct leukemia subgroups (Table S6). All performed statistical tests were two tailed, and no corrections for multiple testing were used.

## Results

### SOX11 is overexpressed in acute lymphoblastic leukemias

**Figure 1:**
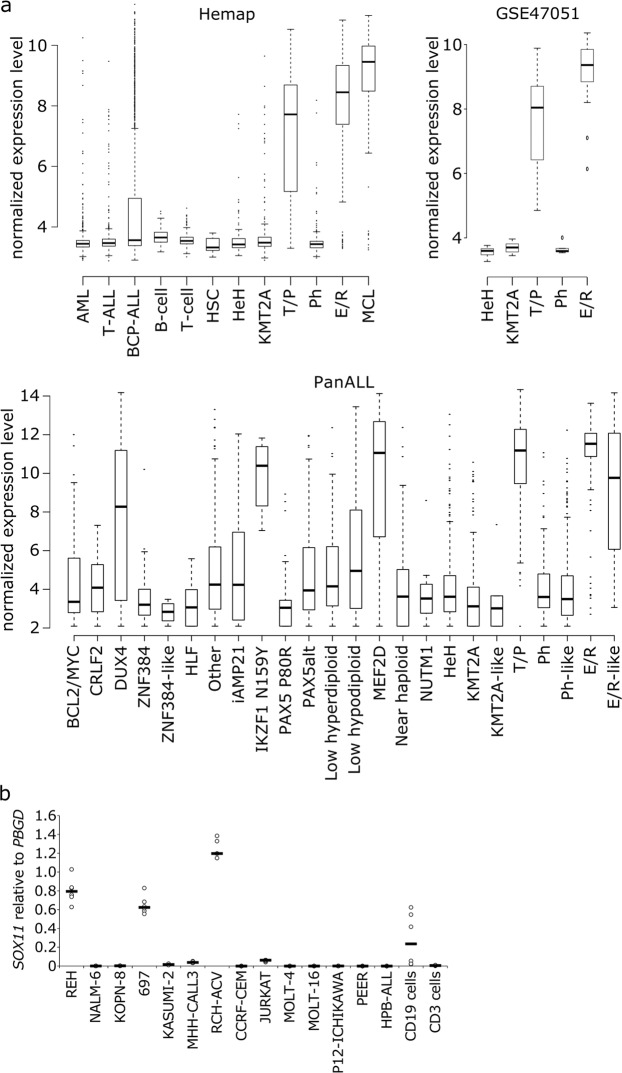
*SOX11* expression in hematological malignancies. **(a)** Expression boxplots of *SOX11* in healthy cells, leukemias, and mantle cell lymphoma. Data sources: Hemap microarray dataset^34^, GSE47051^35^, and the PanALL study^36^. See Supplementary Information for more details. **(b)** *SOX11* expression in ALL cell lines and healthy B- and T-cells, as measured by RT-qPCR (N = 2, black lines indicate the median). ALL, acute lymphoblastic leukemia; AML, acute myeloid leukemia; BCL2/MYC, BCL2/MYC rearranged; BCP-ALL, B-cell precursor ALL; CRLF2, CRLF2 (non-Ph-like); DUX4, DUX4 rearranged; E/R, ETV6-RUNX1; HeH, high hyperdiploid; HLF, TCF3/TCF4-HLF; HSC, hematopoietic stem cell; iAMP21, intrachromosomal amplification of chromosome 21; IKZF1 N159Y, IKZF1 missense alteration encoding p.Asn159Tyr; KMT2A, KMT2A rearranged; MCL, mantle cell lymphoma; MEF2D, MEF2D rearranged; NUTM1, NUTM1 rearranged; PAX5alt, PAX5 alterations; PAX5 P80R, PAX5 p.Pro80Arg (P80R) alteration; Ph, Philadelphia chromosome; T/P, TCF3-PBX1; ZNF384, ZNF384 rearranged. Reproduced with permission^66^.

Derailed differentiation and abnormal proliferation of B-cells are thought to underlie the genesis of BCP-ALL. As TFs are key cell differentiation drivers, we sought to identify aberrantly expressed TFs in BCP-ALL. We utilized a large, curated dataset of microarray-based gene expression profiles retrieved from GEO^34^. This dataset comprises a total of 9,544 hematological gene expression profiles, including 4,418 leukemias, 428 healthy controls, and 862 cell lines. Among the genes with altered expression, TF *SOX11* showed prominent expression in MCL and in the E/R and T/P subtypes of BCP-ALL (Fig. 1a). Compared with healthy hematopoietic cells, *SOX11* had a 4.7- and 4-fold higher expression in the E/R and T/P subtypes, respectively, with an expression comparable to that in MCL (Fig. 1a). A similar subtype-specific expression of *SOX11* was replicated in two additional ALL gene expression datasets (Fig. 1a). Interestingly, the PanALL study revealed that *SOX11* expression is also elevated in novel subtypes, such as E/R-like, IKZF1 N159Y (*IKZF1* missense alteration encoding p.Asn159Tyr), MEF2D rearrangement, and DUX4 rearrangement.

As SOX11 belongs to the SoxC family of TFs, we investigated the expression of the two other members of this family. *SOX4*, which has been reported to affect survival, progression, and proliferation in BCP-ALL^48^, was highly expressed in BCP-ALL and T-ALL, but it showed no subtype-specific expression pattern. The expression of *SOX12* did not vary markedly between the studied subtypes (Fig. S1a).

A subtype-specific expression pattern was also present in BCP-ALL cell lines, as REH cells, representing the E/R subtype, and RCH-ACV and 697 cells, representing the T/P subtype, showed an increased expression of *SOX11* by RT-qPCR (Fig. 1b). Neither knockdown of *E/R* in REH cells nor their overexpression in NALM-6 cells had any effect on *SOX11* expression levels (Fig. S2a).

### Immunohistochemical analysis of SOX11 expression in ALL

**Figure 2:**
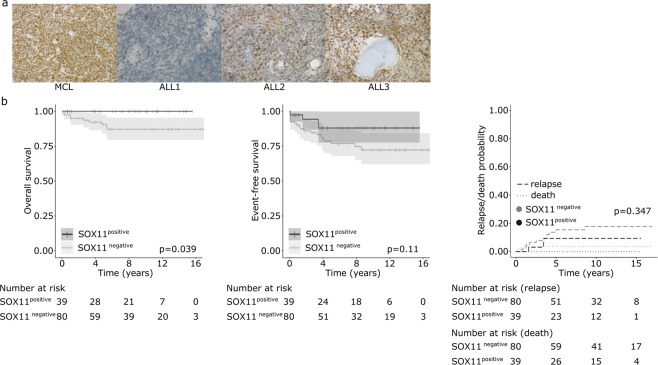
SOX11 protein expression and impact on the outcome in BCP-ALL. **(a)** Expression of SOX11 protein by immunohistochemistry. Mantle cell lymphoma (MCL), a strongly positive case for SOX11 (400×); ALL1, a negative B-ALL case (400×); ALL2, a positive B-ALL case (400×); ALL3, a strongly positive B-ALL case (400×). **(b)** Kaplan–Meier survival curves and Log-rank p-values for OS, EFS, and RFS in the SOX11-positive (high) and -negative (low) groups. Reproduced with permission^66^.

In order to confirm the SOX11 expression at the protein level and relate it to clinical features, we collected a retrospective cohort of 119 B-ALL cases with available bone marrow biopsies and associated clinical data (Table 1). We performed immunohistochemical staining of the biopsy samples by using a SOX11-specific antibody. Staining intensity was graded from 0 to 2, with 0 marking negative, 1 positive, and 2 strongly positive samples. Eighty out of 119 primary B-ALL bone marrow samples stained negative for SOX11, whereas 34 cases were positive and 5 were strongly positive (Fig. 2a and Table 2). A total of 29/39 (74.4%) of the positively staining B-ALL cases represented either the E/R or T/P subtype. A statistically significant association was observed with the E/R subtype (p-value < 0.001) but not with the T/P subtype (likely because of the low number of cases: N = 4, 3 positive cases). Cases with either *KMT2A* rearrangement or the Philadelphia chromosome (*BCR-ABL1-*translocation) did not express the SOX11 protein, and the majority of hyperdiploid cases were also SOX11 negative. Additionally, seven T-ALL cases included in the whole ALL patient cohort were all negative.

**Table 1:** Summary of clinical characteristics of B-ALL patients.

	WHO Subtype								Total/Combined
	Burkitt	NOS	Ph	KMT2A	ETV6-RUNX1	Hyper-diploid	Hypo-diploid	TCF3-PBX1	
N of cases	2	42	2	5	33	30	1	4	119
Age, Md (min/max)	12.7	8.1	10.1	1.3	4	3.7	4.1	7.4	4.3 (0.9/17.6)
WBC count (10^9^/l), Md (min/max)	18.9	6.7	156.3	109.7	7	7.1	1.9	43.4	7.1 (1/311)
Deceased	2	6	1	0	1	1	0	0	9
Relapse	0	7	1	0	3	3	0	1	15
CNS disease	0	2	1	0	1	2	0	0	6
Resistant disease	0	3	0	0	1	0	0	0	4
MRD (%) at EOI, Md (min/max)	0	0.05	1.75	0	0.06	0	0	0	0.02 (0/44)
Follow-up (years), Md (min/max)	7.5	9.7	8.4	4	7.1	9.2	11.5	7.6	8.2 (0.1/17)

CNS, central nervous system; EOI, end of induction therapy; KMT2A, KMT2A rearrangement; Max, maximum; Md, median; Min, minimum; MRD, minimal residual disease; NOS, not otherwise specified; Ph, Philadelphia chromosome; WBC, white blood cell.

**Table 2:** Summary of SOX11 protein expression by immunohistochemical staining in B-ALL.

SOX11 IHC	WHO Subtype								All cases
	Burkitt	NOS	Ph	KMT2A	ETV6-RUNX1	Hyper-diploid	Hypo-diploid	TCF3-PBX1	
Negative	1	35	2	5	7	29	0	1	80
Positive	1	6	0	0	24	0	1	2	34
Strong positive	0	1	0	0	2	1	0	1	5
Total	2	42	2	5	33	30	1	4	119

IHC, immunohistochemistry; KMT2A, KMT2A rearrangement; NOS, not otherwise specified; Ph, Philadelphia chromosome.

### SOX11 expression is associated with a favorable outcome

We next sought to evaluate the clinical significance of SOX11 expression in B-ALL (N = 119). The overall survival (OS) was better in the SOX11-positive group, and no deaths occurred among SOX11-positive cases (p = 0.039) (Fig. 2b). The EFS and relapse-free survival (RFS) adjusted for the competing event (death) showed similar trends but did not reach statistical significance (Fig. 2b). SOX11 positivity was not associated with good early therapy response, as measured by a minimal residual disease below 0.1% at the end of induction therapy (OR = 0.54, 95% CI 0.22, 1.28, p = 0.17). In multivariate analysis of EFS with covariates (age, WBC, MRD and subtypes), a positive immunohistochemical staining for SOX11 protein showed a favorable trend (Table 3).

**Table 3:** Multivariate and univariate analyses of the event-free survival based on expression level of SOX11 protein in pediatric B-ALL.

	N	MULTIVARIATE			UNIVARIATE		
		HR	95% CI	*p-value*	HR	95% CI	*p-value*
**Age (years)**							
≥1 and ≤10	93	1.00*			1.00*		
<1 and >10	26	0.87	0.31–2.38	0.79	1.27	0.50–3.23	0.61
**WBC count (10**^**9**^**/l)**							
<50	101	1.00*			1.00*		
≥50	18	0.55	0.16–1.92	0.35	0.90	0.27–3.04	0.87
**Subtype**							
Other B-ALL	56	1.00*			1.00*		
High hyperdiploidy	30	0.27	0.07–1.00	0.05	0.45	0.15–1.36	0.16
ETV6-RUNX1	33	0.72	0.18–2.83	0.64	0.50	0.17–1.51	0.22
**SOX11 expression**							
negative	80	1.00*			1.00*		
positive	39	0.37	0.10–1.43	0.15	0.43	0.15–1.26	0.12
**MRD at EOI**							
<0.1%	78	1.00*			1.00*		
≥0.1%	37	1.70	0.68–4.24	0.26	2.18	0.94–5.06	0.07

Cox proportional hazards regression calculated for known risk factors. SOX11 expression was treated as a binary variable. 95% CI, 95% confidence interval; B-ALL, B-cell acute lymphoblastic leukemia; HR, hazard ratio; WBC, white blood cell. *Marks reference groups of each categorical variable.

When SOX11 immunostaining positivity was analyzed separately within the E/R subtype, SOX11-positive cases had a better OS (Log-rank test p = 0.004), but EFS did not show a statistically significant difference with a hazard ratio of 0.67 (95% CI 0.07, 6.43).

**Figure 3:**
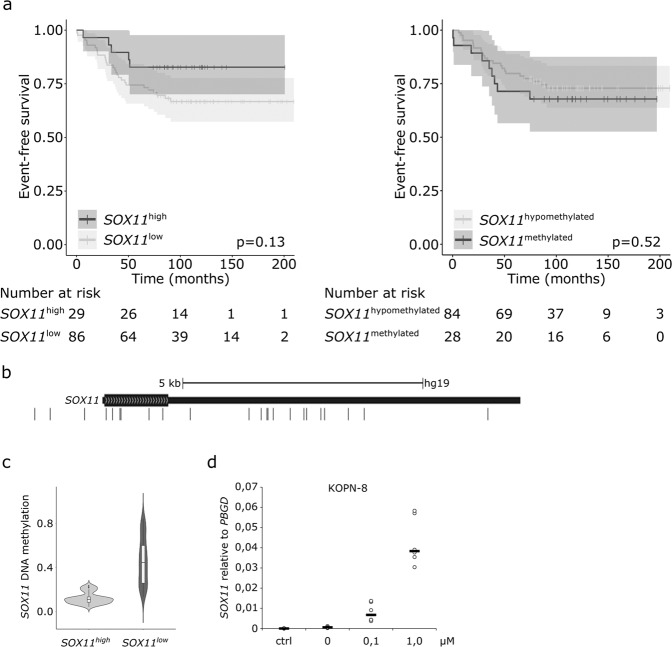
Survival analysis and methylation status of cases with either a high or low *SOX11* mRNA expression. **(a)** Kaplan–Meier survival curves and p-values of Log-rank test for EFS in patients with a low or high expression of *SOX11* and in patients with a low or high methylation of the *SOX11* gene locus. **(b)** CpG sites at the *SOX11* locus in chromosome 2^35^. **(c)** DNA methylation at the *SOX11* gene locus among patients with either a low or high expression of *SOX11*. **(d)** Effect of decitabine treatment on *SOX11* mRNA expression in the KOPN-8 cell line. Reproduced with permission^66^.

We replicated the survival findings in another dataset. Transcriptomic data from a patient cohort comprising 115 BCP-ALL cases were analyzed for *SOX11* expression and patients’ survival status^46^. Figure 3a shows that a high *SOX11* mRNA expression was associated with a favorable trend in EFS analysis.

### *SOX11* overexpression is associated with DNA hypomethylation

We next investigated the biology behind the increased *SOX11* expression in leukemia. Neither primary transcription (GRO-seq, N = 8) nor whole-genome sequencing (WGS, N = 8) of the *SOX11* gene in BCP-ALL cases revealed any aberrant enhancer activity or somatic mutations, respectively, in the *SOX11* gene or nearby regions (data not shown), prompting us to look for other mechanisms. In MCL, hypomethylation (partly) drives the increased *SOX11* expression^30^. We utilized the above-mentioned BCP-ALL patient cohort with readily available genome-wide CpG methylation data^35,46^. Altogether, 23 CpG sites were located within the *SOX11* gene locus, and a strong pattern of DNA hypomethylation was seen in patients with a high *SOX11* mRNA expression (Fig. 3b,c). Nevertheless, DNA hypomethylation of the CpG sites at the *SOX11* locus was not associated with a better EFS (Fig. 3a, right panel). We also tested whether a methyltransferase inhibitor, decitabine, could reverse *SOX11* expression in leukemia cell lines. After 72 h of decitabine treatment, a marked increase in *SOX11* expression was observed in a concentration-dependent manner in KOPN-8 and REH cells (Figs. 3d and S2b).

### Knockdown of *SOX11* alters gene sets related to cell development, motility, and drug response pathways

**Figure 4:**
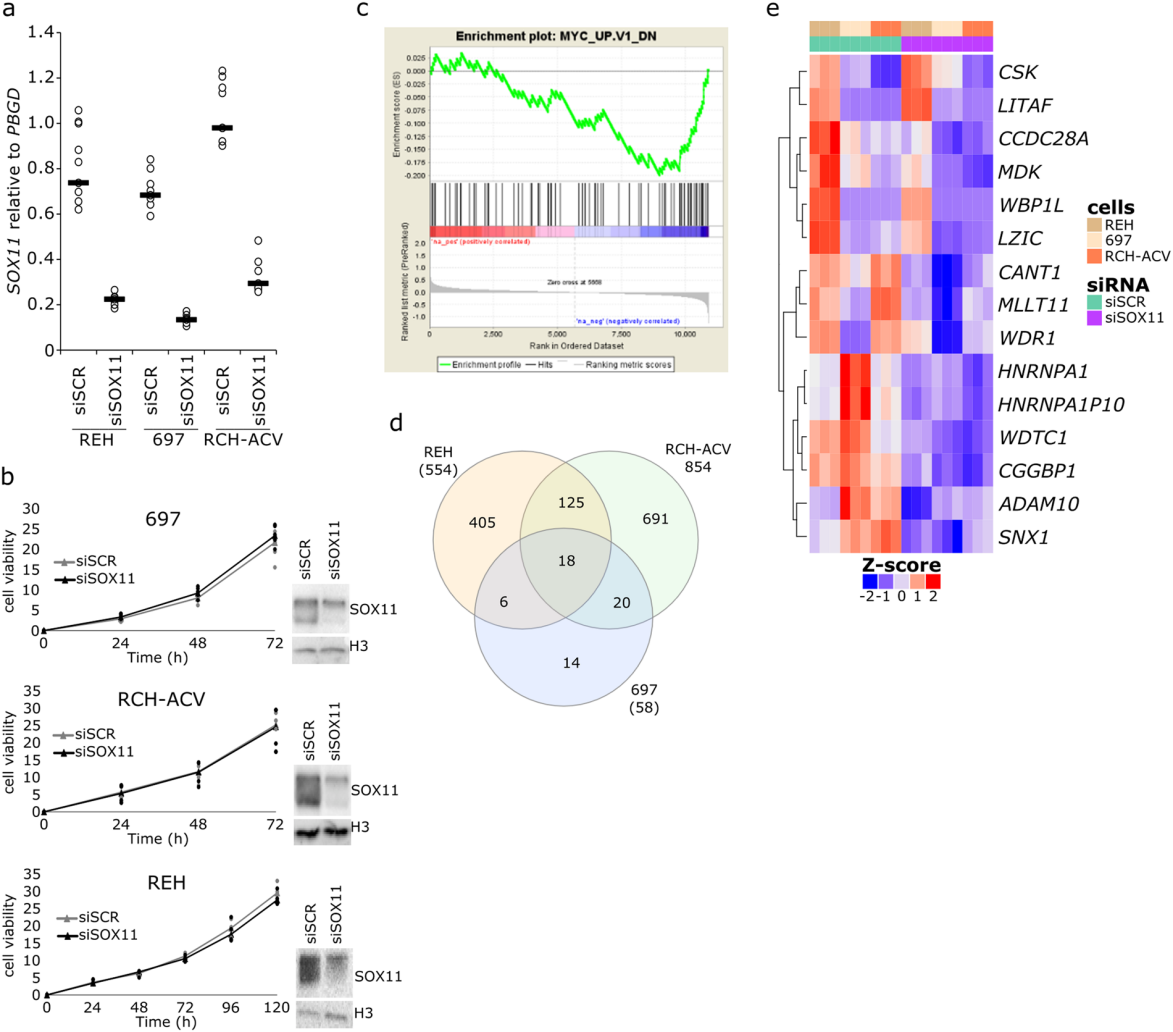
Cell viability and transcriptomic changes after knockdown of *SOX11*. **(a)** Expression level of *SOX11* after knockdown, as analyzed by RT-qPCR (N = 3, black lines indicate the median). **(b)** Knockdown of *SOX11* caused no evident changes in cell viability. Curves are drawn from the biological replicates using the median value at given time points. 697 and RCH-ACV cells represent the T/P subtype, and REH cells represent the E/R subtype. *SOX11* knockdown was confirmed by Western blotting, and cell viability assessments were conducted with the AlamarBlue assay. Measured absorbance intensities are reported as x10^3^ (697 N = 6; RCH-ACV and REH N = 4). Western blot gel figures are cropped per cell line from the original blot figures shown in Fig. S3. **(c)** *SOX11* knockdown in 697 cells caused downregulation of genes that are known to be altered after MYC upregulation. **(d)** Venn diagram of differentially expressed genes in the REH, 697, and RCH-ACV cell lines after knockdown of *SOX11* (adjusted p-value < 0.05). **(e)** Heatmap of 15 concordantly differentially expressed genes in all three cell lines after knockdown of *SOX11*. Reproduced with permission^66^.

We next silenced *SOX11* expression in three cell lines that overexpress *SOX11* (REH, RCH-ACV, and 697) by using siRNA oligos. Figure 4a,b show that *SOX11* expression was decreased to 20–40% at both the mRNA and protein levels compared with scrambled siRNA-transfected cells. Cell viability and proliferation assays did not demonstrate any significant changes (Figs. 4b and S4), and *SOX11* knockdown did not have any impact on sensitivity to known leukemia drugs, such as dexamethasone, prednisolone, vincristine, and asparaginase (Fig. S5).

As SOX11 is a TF and it regulates gene expression, we measured changes in gene expression after *SOX11* knockdown by using RNA-seq (GSE123943). Three biological replicates were used for each cell line (REH, RCH-ACV, and 697), and the data were analyzed using the R package EdgeR. In GO annotations, many of the altered terms were related to cell migration, adhesion, and differentiation (Fig. S6 and Supplementary Dataset). On the other hand, GSEA implied the altered expression of MYC and EF2 target genes (Figs. 4c and S6). *SOX11* knockdown did not have a significant effect on the expression of other SoxC family members, such as *SOX4* and *SOX12* (Fig. S7).

The Venn diagram in Fig. 4d shows that 18 genes were differentially expressed in all of the three cell lines after *SOX11* knockdown (with an adjusted p-value < 0.05). Of these genes, 15 were concordantly down-regulated in all three cell lines and are shown in the heatmap (Fig. 4e, Supplementary Dataset). *SOX11* knockdown led to the downregulation of the *WD Repeat Domain 1* (*WDR1*) gene, which is involved in the remodeling of the actin cytoskeleton, regulation of cell migration, motility of neutrophils, and maturation of megakaryocytes^49,50^. Another gene down-regulated by *SOX11* knockdown is *Midkine* (*MDK*), a secreted growth factor that promotes cell migration and growth and is associated with an adverse prognosis in ALL possibly via increased drug resistance^51,52^. *WW Domain Binding Protein 1 Like* (*WBP1L*), also known as *OPAL1* (outcome predictor for acute leukemia 1), which was recently identified as a direct target of ETV6 in ALL^53^, was down-regulated by *SOX11* silencing. Previous reports on *WBP1L*’s association with a favorable prognosis have since been refuted^54^. However, *WBP1L* expression had a 2.8-fold increase in the E/R subtype^55^, which coincides with the overexpression of *SOX11* in the same subtype and may suggest co-regulation. *MLLT11* is involved in lymphoid regulation and is a known partner gene in rare leukemia translocations^56,57^. Similarly, *Coiled-coil domain containing 28* *A* (*CCDC28A*) is a fusion partner to NUP98 in AML^58^. *CANT1* is a calcium-dependent nucleotidase involved in pyrimidine metabolism whose regulation by SOX11 could be related with drug metabolism (cytarabine) and therapy response in ALL^59^. The *lipopolysaccharide-induced TNF factor* (*LITAF*) has been suggested to sensitize leukemia cells to chemotherapeutic drugs, especially in cells with a lower expression of *LITAF*^60^. Both *LITAF* and *Sorting nexin 1* (*SNX1*) are involved in endosomal trafficking and regulation of cell-surface receptor signaling^61,62^. Taken together, *SOX11* knockdown leads to alterations in genes and cellular processes related to leukemia cell motility, adhesion, differentiation, and drug response.

### SOX11 protein can be detected by flow cytometry

**Figure 5:**
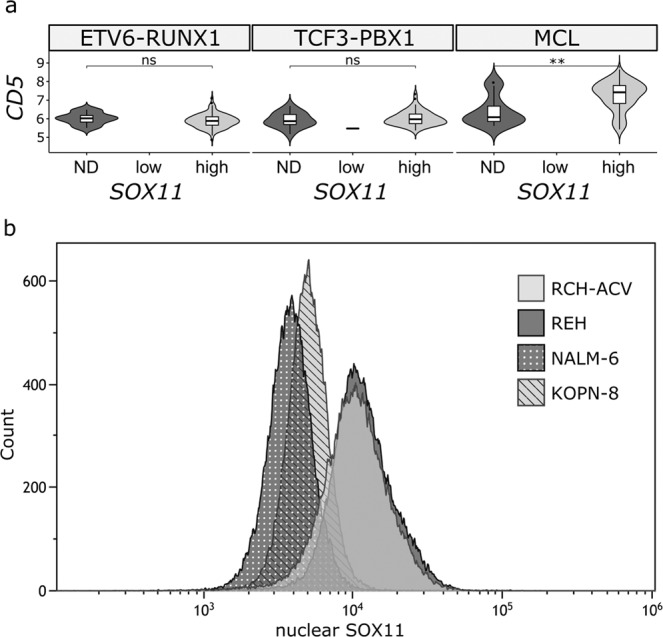
Detection of SOX11-positive cells by flow cytometry in BCP-ALL. **(a)** Correlation of *CD5* expression with *SOX11* mRNA expression in the E/R and T/P subtypes of B-ALL and MCL^34^. *SOX11* expression was categorized into three groups, not detected (ND), low and not detected (ND). **(b)** Flow cytometry analysis of nuclear SOX11 expression in cell lines with either a low (NALM-6, KOPN-8) or high (REH, RCH-ACV) expression of SOX11. ND, not detected, MCL, mantle cell lymphoma. Reproduced with permission^66^.

Finally, we searched for flow cytometry markers that could serve as surrogates of SOX11 positivity. In MCL, the surface expression of CD5 is correlated with SOX11 positivity^22^, but in our immunohistochemical staining and microarray dataset^34^, no positive correlation was observed in leukemias (Fig. 5a). Alternative surrogate markers were searched for among the routinely studied cell surface proteins, but none were associated with SOX11 positivity (data not shown). Therefore, we explored the suitability of SOX11 antibodies to discriminate SOX11-positive cases by flow cytometry. Figure 5b shows that the intensity of SOX11 antibody staining could readily separate the high expressors (REH and RCH-ACV) from the low expressors (NALM-6 and KOPN-8) in leukemia cell lines, suggesting that SOX11 antibodies could possibly be used as a biomarker in the future.

## Discussion

BCP-ALL is caused by a relatively small number of genetic mutations that impede normal B-cell differentiation, entail self-renewal capacity, and increase proliferative activity. This is evident in transcriptional programs that govern leukemic B-cells. We report here that SOX11, a developmental and neuronal TF^6^, is overexpressed in the E/R and T/P subtypes of BCP-ALL and also in novel E/R-like, IKZF1 N159Y, MEF2D rearrangement, and DUX4 rearrangement subtypes. A high expression is associated with DNA hypomethylation and a favorable clinical outcome. The results suggest that a SOX11-associated transcriptional program is related with a less-aggressive disease and indicates that SOX11 warrants further study as a biomarker for low-risk ALL patients.

We observed the high expression of *SOX11* in the E/R and T/P subtypes of BCP-ALL in three separate gene expression datasets at the mRNA level^34–36^ and confirmed this association at the protein level by immunohistochemistry, confirming previously published data^8,13,29,30^ and adding further evidence at the protein level. We showed that the expression may be regulated epigenetically, that is, by hypomethylation of DNA at the *SOX11* locus, similar to what was earlier reported for MCL and five cases of BCP-ALL by Vegliante *et al*.^30^. We did not have material available for epigenetics studies, such as histone modifications, as these could also have a role in *SOX11* regulation^30^. Direct manipulation of the *E/R* fusion did not have any impact on *SOX11* expression, suggesting that regulation is indirect.

An interesting finding relates to the clinical significance of SOX11 expression in BCP-ALL, as we observed a better OS in the SOX11-positive cases. A similar trend was also seen in EFS, RFS, and early therapy response. This finding was supported (similar trend) in another dataset with transcriptome expression profiles^46^. In the subgroup analysis of E/R cases, SOX11 positivity retained its prognostic significance, suggesting that (a high) SOX11 expression could possibly be utilized as a biomarker for cases with a very good prognosis. It is noteworthy that our immunohistochemical staining series spans almost two decades, and patients have been treated using several distinct NOPHO ALL chemotherapy protocols^63,64^. As the most recent protocols have conferred the best survival results [63, trying to replicate these findings in the most recent protocols is necessary in the future.

To aid in the screening of SOX11 positivity at diagnosis, we also successfully tested a flow cytometry-based assay in cell lines. SOX11 inclusion into the flow cytometry panel would be convenient compared with immunohistochemical staining of bone marrow biopsies, which is slow and not routinely done in all treatment centers.

*SOX11* knockdown did not markedly influence cell viability or proliferation, nor did it affect chemotherapy sensitivity. In MCL, conflicting reports have been made about the effect of knockdown or *SOX11* overexpression on cell proliferation and tumor growth^16–23^. As cell viability measurement is a relatively insensitive assay, we performed transcriptional profiling of *SOX11* knockdown cells by using RNA-seq and noticed changes in the genes associated with cell migration, adhesion, oxidative phosphorylation, hypoxia, glycolysis, and differentiation, which could explain the association of SOX11 with favorable clinical outcomes. Notably, the changes observed were mostly mild to moderate. There were only few overlapping genes with previous profiling studies in both pro-B-cells^18^ and MCL cells^16,17,20,21,65^. For example, we did not see marked changes in the expression of either *PAX5*, as seen in MCL^21^, or *Id1* and *Tal1* in pro-B-cells^18^. Interestingly, *MDK*, which is involved in cell migration and growth, was downregulated by *SOX11* knockdown in leukemia cell lines here and in an MCL cell line Z138^16^.

In conclusion, the association of SOX11 expression with a favorable prognosis invites further studies to confirm its prognostic value and applicability as a part of the diagnostic workup.

## Supplementary information

Supplementary Information.

Dataset 1.

## Data Availability

The datasets generated and analyzed in the current study are available in the GEO repository, GSE47051 (https://www.ncbi.nlm.nih.gov/geo/query/acc.cgi?acc=GSE47051), GSE123943 (https://www.ncbi.nlm.nih.gov/geo/query/acc.cgi?acc=GSE123943), and GSE49031 (https://www.ncbi.nlm.nih.gov/geo/query/acc.cgi?acc=GSE49031), or are included in the article or supplementary files. RNA-seq data of the patient samples^46^ are not publicly available, as the patient/parent consent does not cover depositing data into repositories; however, they are available from the authors upon reasonable request.
