# Supplementary material for: Clinicopathological features and prognostic value of SOX11 in childhood acute lymphoblastic leukemia
Source: Sci Rep. 2020 Feb 6;10:2043. doi: 10.1038/s41598-020-58970-z (PMC7005266; doi:10.1038/s41598-020-58970-z)
Supplement: Supplementary file 1 — Supplementary Information. [file 41598_2020_58970_MOESM1_ESM.pdf]

Clinicopathological features and prognostic value of SOX11 in childhood acute lymphoblastic leukemia

Toni Grönroos†* [1], Artturi Mäkinen† [1,2], Saara Laukkanen [1], Juha Mehtonen [3], Atte Nikkilä [1], Laura Oksa [1], Samuli Rounioja [4], Yanara Marincevic-Zuniga [5], Jessica Nordlund [5], Virva Pohjolainen [2], Timo Paavonen [2,6], Merja Heinäniemi [3], Olli Lohi [1,7]

1. Tampere Center for Child Health Research, Faculty of Medicine and Health Technology, Tampere University, Tampere Finland

2. Fimlab Laboratories, Department of Pathology, Tampere University Hospital, Tampere, Finland

3. Institute of Biomedicine, School of Medicine, University of Eastern Finland, Kuopio, Finland

4. Fimlab Laboratories, Department of Hematology, Tampere University Hospital, Tampere, Finland

5. Department of Medical Sciences, Molecular Medicine and Science for Life Laboratory, Uppsala University, Uppsala, Sweden

6. Department of Pathology, Faculty of Medicine and Health Technology, Tampere University, Tampere, Finland

7. Department of Pediatrics, Tampere University Hospital, Tampere, Finland

†These authors contributed equally to this work

* Corresponding author: Toni Grönroos

Postal address: Tampere University, Arvo Ylpön katu 34, Arvo Building, 33520, Tampere, Finland

phone: +358 50 3186 249
e-mail: toni.gronroos@tuni.fi

Supplementary Table 1. Dataset sample sizes

Group	Hemap	GSE47051	PanALL
T-ALL	385	30	0
HeH	151	18	279
Low hyperdiploid	0	0	51
KMT2A	301	5	136
KMT2A-like	0	0	5
TCF3-PBX1	81	6	78
ETV6-RUNX1	171	16	187
ETV6-RUNX1-like	0	0	42
Ph	137	5	123
Ph-like	0	0	359
B-cell	75	0	0
HSC	21	0	0
T-cell	247	0	0
BCP-ALL	1304	0	0
AML	1713	0	0
MCL	100	0	0
BCL2/MYC	0	0	18
CRLF2	0	0	16
DUX4	0	0	106
HLF	0	0	9
iAMP21	0	0	40
IKZF1 N159Y	0	0	8
Low hypodiploid	0	0	78
MEF2D	0	0	43
Near haploid	0	0	29
NUTM1	0	0	11
Other	0	0	125
PAX5 P80R	0	0	44
PAX5alt	0	0	148
ZNF384	0	0	49
ZNF384-like	0	0	4

ALL, acute lymphoblastic leukemia; AML, acute myeloid leukemia; BCL2/MYC, BCL2/MYC rearranged; BCP-ALL, B-cell precursor ALL; CRLF2, CRLF2 (non-Ph-like); DUX4, DUX4 rearranged; HeH, high hyperdiploid; HLF, TCF3/TCF4-HLF; HSC, hematopoietic stem cell; iAMP21, intrachromosomal amplification of chromosome 21; IKZF1 N159Y, IKZF1 missense alteration encoding p.Asn159Tyr; KMT2A, KMT2A rearranged; MCL, mantle cell lymphoma; MEF2D, MEF2D rearranged; NUTM1, NUTM1 rearranged; PAX5alt, PAX5 alterations; PAX5 P80R, PAX5 p.Pro80Arg (P80R) alteration; Ph, Philadelphia chromosome; T-ALL, T-cell ALL; ZNF384, ZNF384 rearranged.

Supplementary Table 2. RT-qPCR primer sequences

Primer	Sequence 5'→ 3'
SOX11 forward	CGGTCAAGTGCGTGTTTCTG
SOX11 reverse	CACTTTGGCGACGTTGTAGC
PBGD forward	CGCATCTGGAGTTCAGGAGTA
PBGD reverse	CCAGGATGATGGCACTGA
SOX4 forward	GGCACATCAAGCGACCCATG
SOX4 reverse	CGTTTGCCCAGCCGCTTGG
SOX12 forward	CGAGGACGACGATGAAGACG
SOX12 reverse	GGACCATCCTCCACAGCTC
ETV6-RUNX1 forward	TGCACCCTCTGATCCTGAAC
ETV6-RUNX1 reverse	AACGCCTCGCTCATCTTGC

Supplementary Table 3. Used corticosteroid and chemotherapy concentrations

Cell line	Prednisolone (µM)	Dexamethasone (µM)	Asparaginase (U/ml)	Vincristine (nM)
697	0, 0.01, 0.1, 1	0, 0.01, 0.1, 1	0, 0.001, 0.01, 0,1	0, 0.1, 0.25, 0.5, 1
RCH-ACV	0, 0.01, 0.1, 1	0, 0.01, 0.1, 1	0, 0.001, 0.01, 0.1, 0.5	0, 0.1, 0.25, 0.5, 1, 2
REH	0, 100, 500, 1000	0, 10, 50, 100	0, 0.001, 0.01, 0.1	0, 0.1, 0.25, 0.5, 1

Supplementary Table 4. siRNA sequences

siRNA	Primers 5' → 3'	Reference
siSCR sense	GUUGCGUAGCGUACGUCGCAA	
siSCR antisense	UUGCGACGUACGCUACGCAAC	
siSOX11 sense	GAUAAGAUGUCGUGACGCA	[1]
siSOX11 antisense	UGCGUCACGACAUCUUAUC	[1]

Supplementary Table 5. Transfection protocols

Cell line	Solution	Reaction
697	SG	CA-137
RCH-ACV	SG	CM-137
REH	SF	CA-137

Supplementary Table 6. Kruskal-Wallis H test and Mann-Whitney U test was used to evaluate differential expression of *SOX11* in distinct leukemia subgroups

Mann-Whitney U test (p-value)			
**Groups**	**Hemap**	**GSE47051**	**PanALL**
TCF3-PBX1 vs rest	9.09×10^{-20}	8.63×10^{-2}	1.69×10^{-27}
ETV6-RUNX1 vs rest	3.04×10^{-52}	1.81×10^{-9}	2.09×10^{-78}
	**Kruskal-Wallis H test (p-value)**		
	3.12×10^{-104}	3.16×10^{-7}	5.57×10^{-142}

Library preparation and RNA sequencing

The quality of the total RNA samples was ensured with Advanced Analytical Fragment Analyzer. Library preparation was done according to Illumina TruSeq® Stranded mRNA Sample Preparation Guide (part # 15031047) (Illumina, San Diego, CA, USA). The high quality of the libraries was confirmed with Advanced Analytical Fragment Analyzer and the concentrations of the libraries were quantified with Qubit® Fluorometric Quantitation (Life Technologies). Good quality libraries were exclusively selected and sequenced.

The samples were sequenced with Illumina HiSeq 2500 instrument using Truseq v2 Rapid sequencing chemistry. The samples were normalized and pooled for the automated onboard cluster preparation in HiSeq 2500. The 18 libraries were pooled into one pool and run in 2 lanes. Single-read sequencing with 1 x 50 bp read length was used, followed by 8 + 8 bp dual index run. The base calling was performed using Illumina's standard bcl2fastq2 software, automatic adapter trimming was used.

RNA sequencing and methylation analysis of patient samples

Briefly, strand-specific RNA-seq libraries were prepared from rRNA-depleted RNA using the ScriptSeq V2 Kit and paired-end sequenced to a minimum of 50 bp using a Hiseq2000/2500 or MiSeq instrument (Illumina), producing on average, 46 million read pairs per sample. Sequence reads were aligned to the human 1000 Genomes build 37 (GRCh37) using Tophat 2 (2.0.4) and gene expression levels were normalized to fragments per kilobase per million mapped reads (FPKM) using Cufflinks version 2.2.0.

Briefly, DNA methylation levels were measured using the Infinium HumanMethylation 450k BeadChip assay (Illumina). Intensity signals from the 450k array were converted to methylation beta values (ranging from 0-1) followed by probe filtering and normalization using Peak Based Correction as previously described [2].

References

1. Kuo PY, et al. High-resolution chromatin immunoprecipitation (ChIP) sequencing reveals novel binding targets and prognostic role for SOX11 in mantle cell lymphoma. *Oncogene* 2015; **34**: 1231-1240.

2. Nordlund J, et al. Genome-wide signatures of differential DNA methylation in pediatric acute lymphoblastic leukemia. *Genome Biol* 2013; **14**: r105.

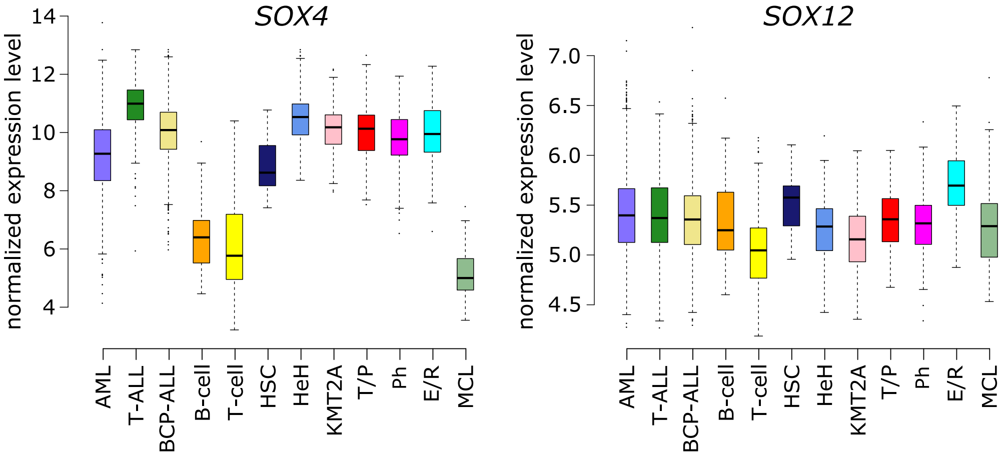

Supplementary Figure 1. *SOX4* and *SOX12* expressions. Expression boxplots of *SOX4* and *SOX12* in healthy cells, leukemias and mantle cell lymphomas. Data source: Combined microarray dataset [1,2]. ALL, acute lymphoblastic leukemia; AML, acute myeloid leukemia; Ph, Philadelphia chromosome; BCP -ALL, B-cell precursor ALL; E/R, ETV6-RUNX1 subtype; HeH, high hyperdiploid subtype; HSC, hematopoietic stem cell; KMT2A, KMT2A rearrangement subtype; MCL, mantle cell lymphoma; T/P TCF3-PBX1 subtype.

References

1. Heinäniemi, M., et al. Transcription-coupled genetic instability marks acute lymphoblastic leukemia structural variation hotspots. *Elife*. 5, e13087 (2016).
2. Pölönen, P., et al. Hemap: An Interactive Online Resource for Characterizing Molecular Phenotypes across Hematologic Malignancies. *Cancer Res*. 79, 2466-2479 (2019).

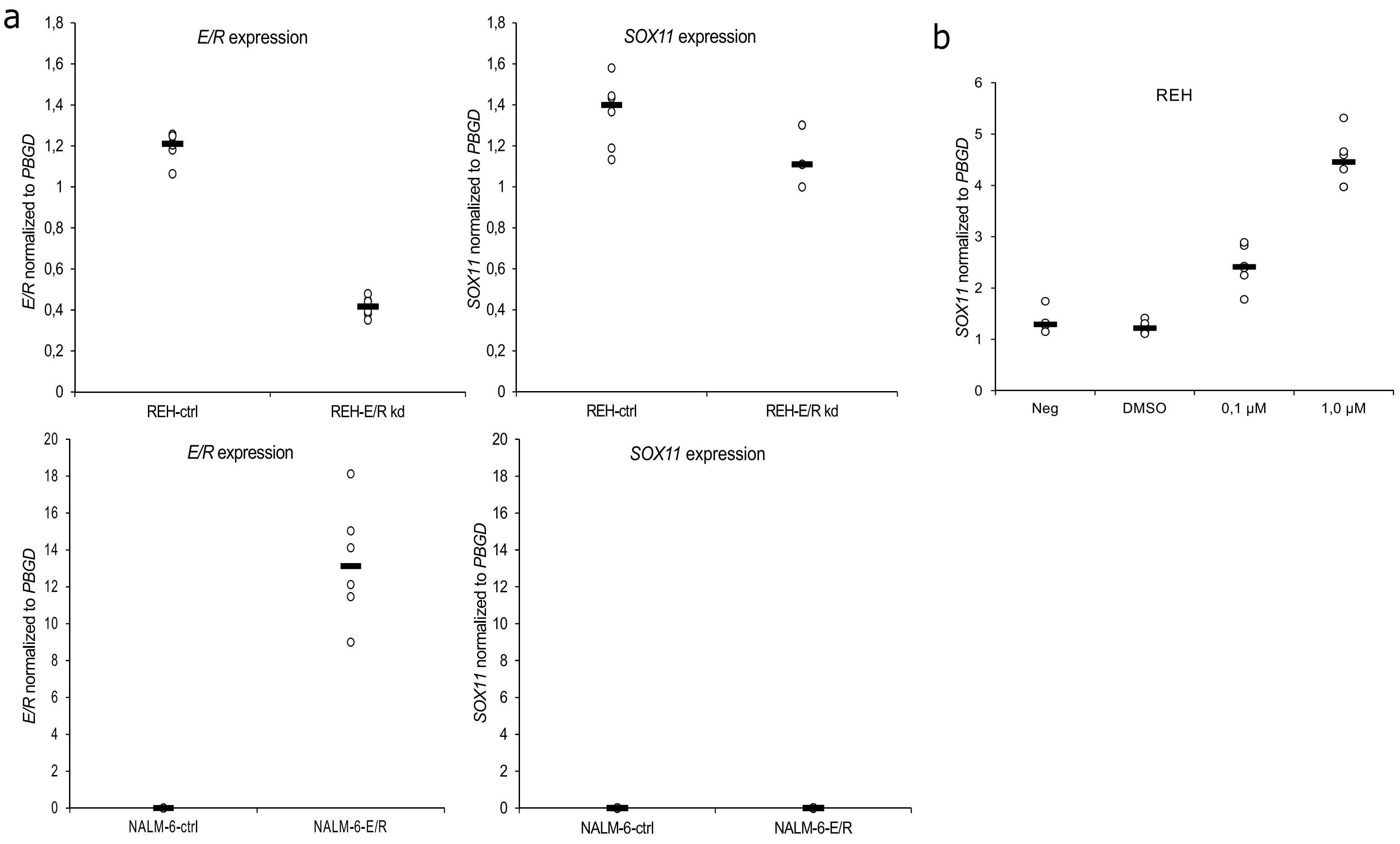

Supplementary Figure 2. *SOX11* expression. a. Changes in *E/R* expression has no marked change on *SOX11* expression (N=2). **b.** Effects of decitabine treatment on *SOX11* expression in REH cell line (N=2).

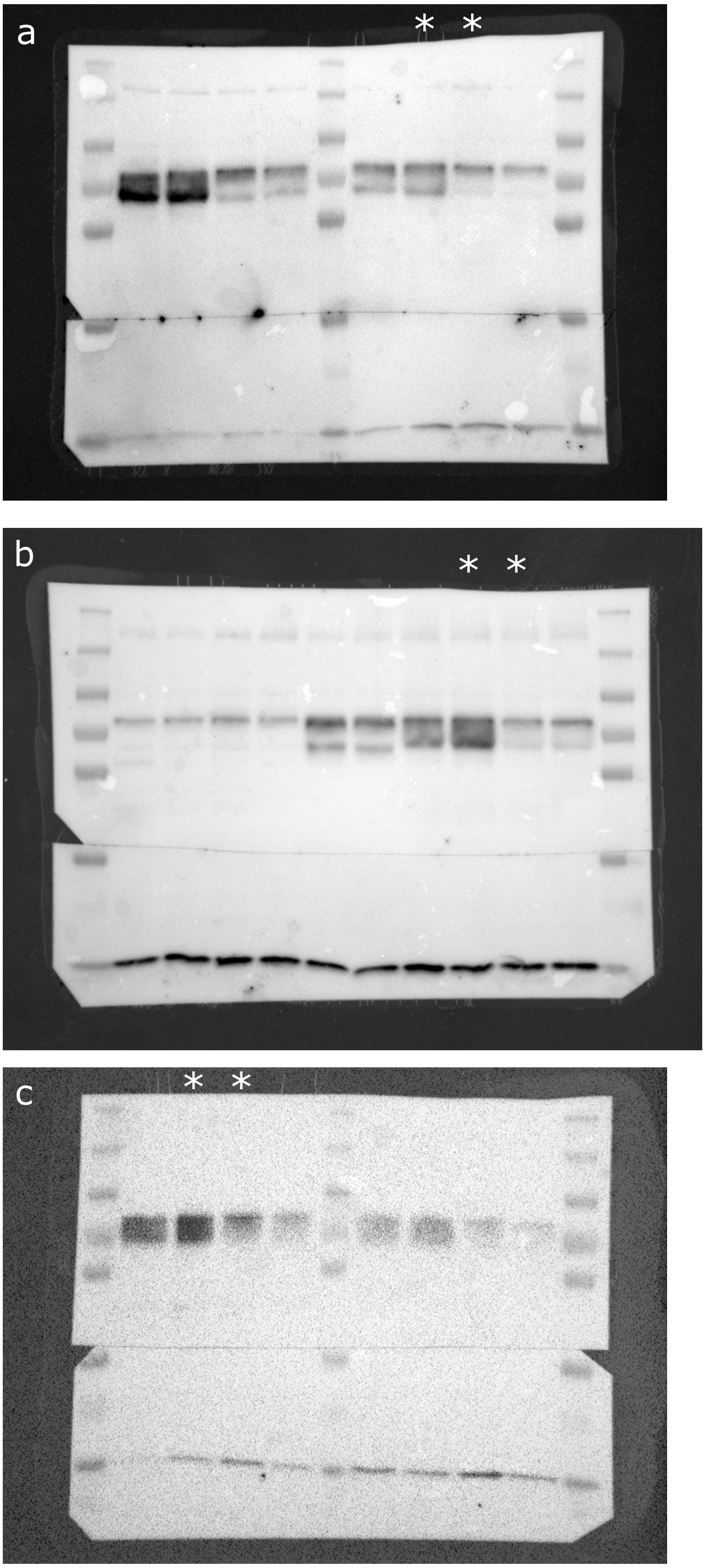

Supplementary Figure 3. Original Western Blots for Figure 4b. a. 697 **b.** RCH-ACV **c.** REH.
*) marks cropped lines for the figure 4 B western blots.

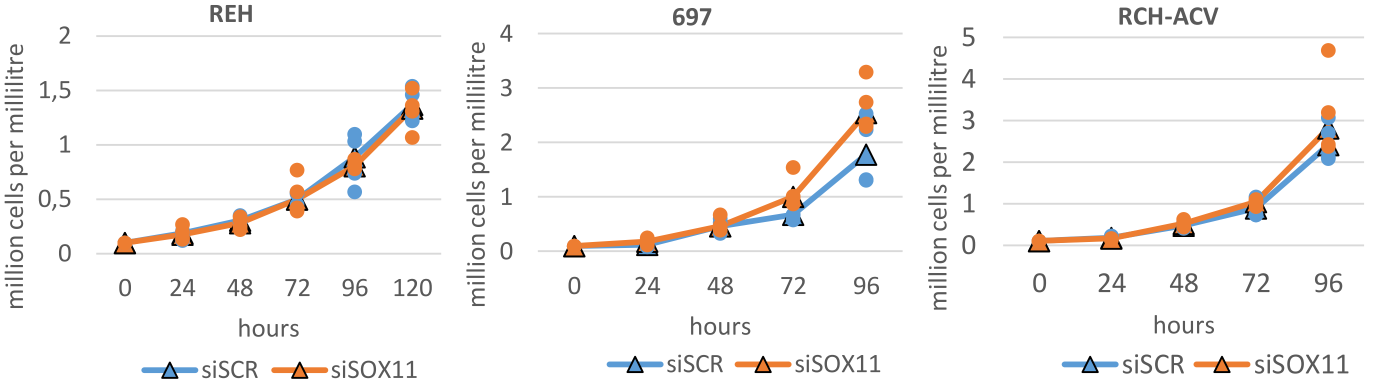

Supplementary Figure 4. Proliferation assay in REH, 697 and RCH-ACV cell lines after *SOX11* knockdown. Proliferation was assayed by counting the cells every 24 hours after the siRNA (siSOX11 = *SOX11* siRNA, siSCR = control siRNA) transfection. *SOX11* knockdown was confirmed with RT-qPCR and N=4.

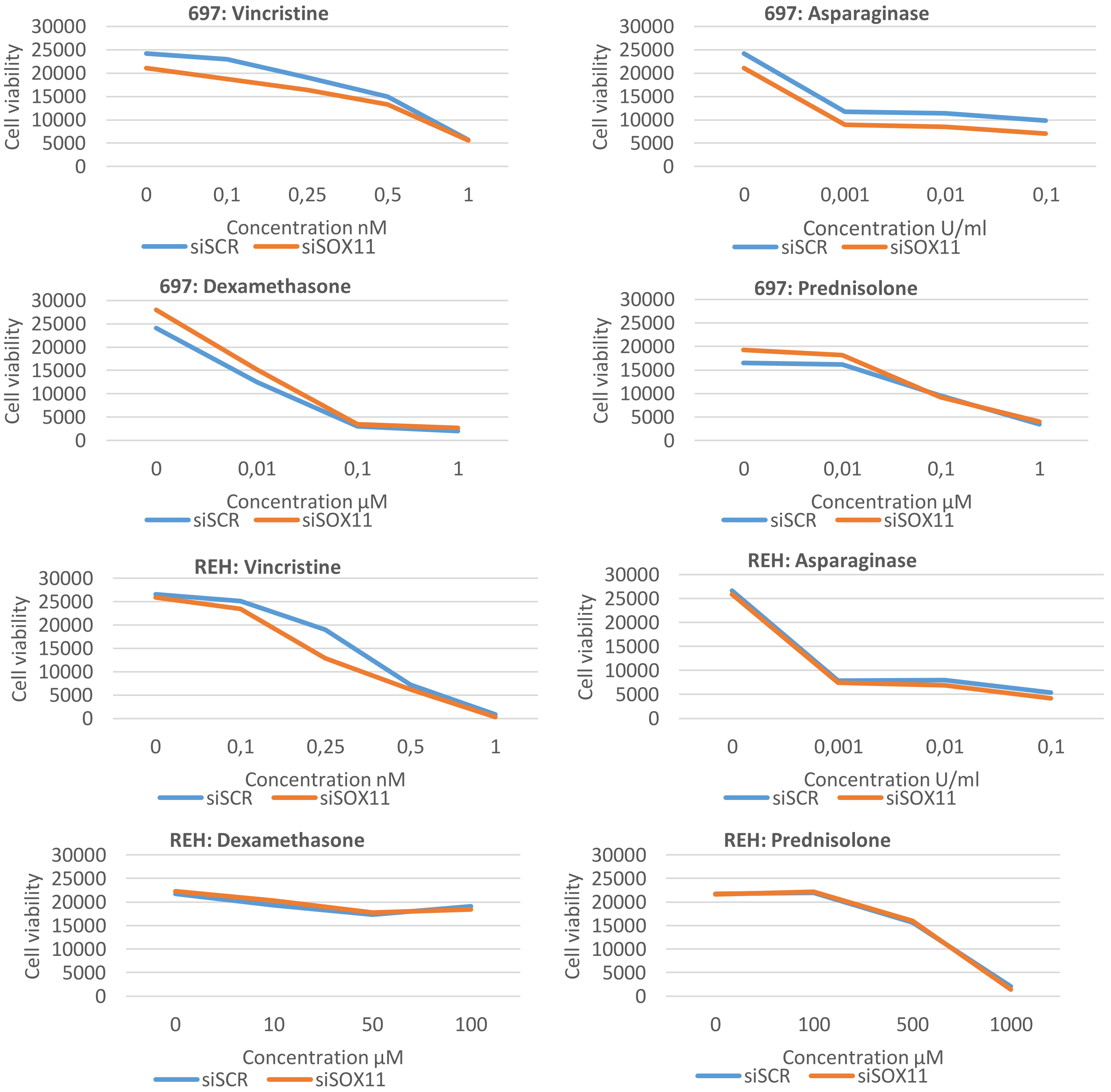

Supplementary Figure 5. Chemotherapy agent and corticosteroid tretments on REH and 697 cell lines. Cell viabilities after siRNA transfection, *SOX11* siRNA (siSOX11) and control siRNA (siSCR) followed by drug treatments (697: 72 hours, REH: 96 hours; N=2). *SOX11* knockdown was confirmed with RT-qPCR and cell viability assessments were carried out with Alamar Blue assay.

a

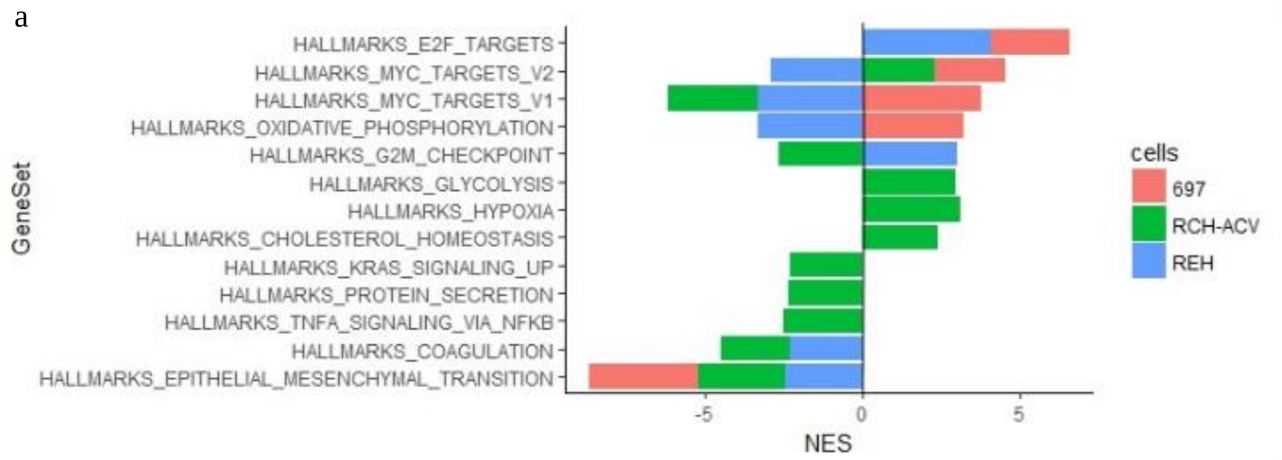

b

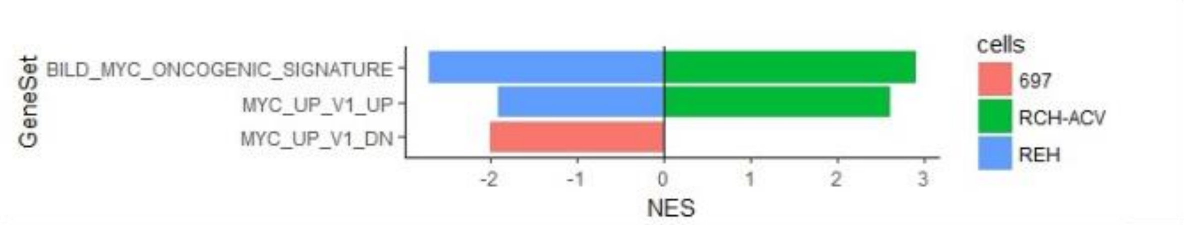

c

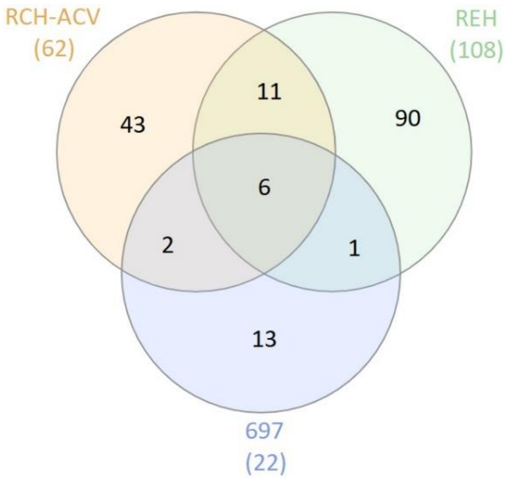

ALL (6)

- GO_HOMOPHILIC_CELL_ADHESION_VIA_PLASMA_MEMBRANE_ADHESION_MOLECULES
- GO_BIOLOGICAL_ADHESION
- GO_CELL_CELL_ADHESION_VIA_PLASMA_MEMBRANE_ADHESION_MOLECULES
- GO_CELL_CELL_ADHESION
- GO_TAXIS
- GO_POSITIVE_REGULATION_OF_CELL_DIFFERENTIATION

RCH-ACV and REH (11)

- GO_REGULATION_OF_MAPK_CASCADE
- GO_NEURON_DIFFERENTIATION
- GO_CELL_MOTILITY
- GO_CELL_MORPHOGENESIS_INVOLVED_IN_DIFFERENTIATION
- GO_POSITIVE_REGULATION_OF_IMMUNE_SYSTEM_PROCESS
- GO_NEURON_PROJECTION_DEVELOPMENT
- GO_IMMUNE_RESPONSE
- GO_REGULATION_OF_MAP_KINASE_ACTIVITY
- GO_MESENCHYMAL_CELL_DIFFERENTIATION
- GO_ACTIN_FILAMENT_BASED_PROCESS
- GO_REGULATION_OF_CELL_ACTIVATION

RCH-ACV and 697 (2)

- GO_REGULATION_OF_ERK1_AND_ERK2_CASCADE
- GO_NEUROLOGICAL_SYSTEM_PROCESS

REH and 697 (1)

- GO_REGULATION_OF_LEUKOCYTE_PROLIFERATION

Supplementary Figure 6. Enrichment of gene sets in 697, RCH-ACV and REH. a. Enrichment of hallmark gene sets retrieved from molecular signature database (MSigDB) in REH, RCH-ACV and 697 cell lines. Adjusted p-value of enrichment for all gene sets < 0.01. **b.** Enrichment of founder gene sets of "MYC targets V2" gene set in REH, RCH-ACV and 697 cell lines. Adjusted p-value of enrichment for all gene sets < 0.02. **c.** Venn diagram of significantly enriched GO terms in *SOX11* siRNA treated REH, RCH-ACV and 697 cell lines and list of GO terms overlapping between cell lines.

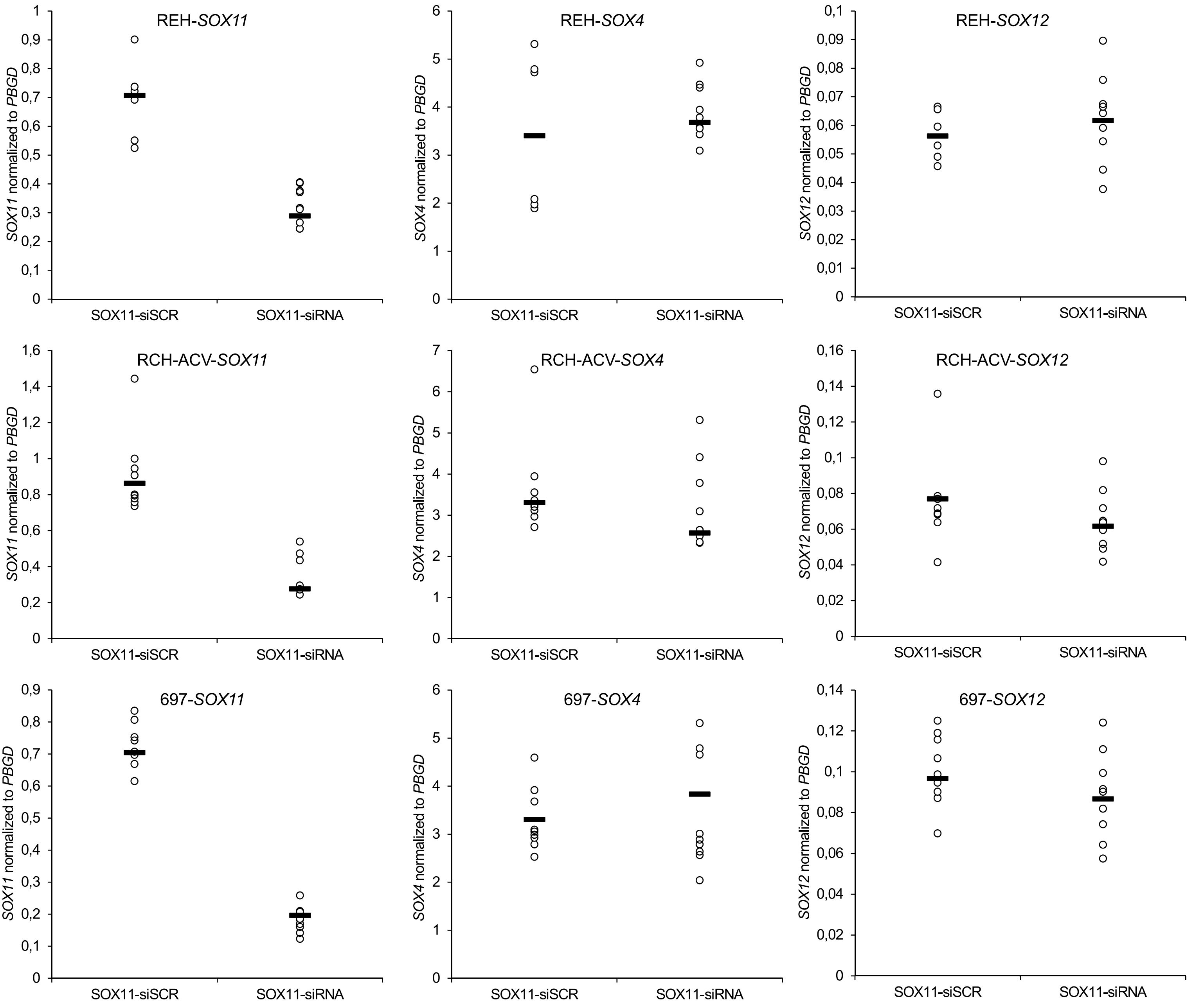

Supplementary Figure 7. SOX4 and SOX12 expressions after SOX11 knockdown. *SOX11* knockdown does not affect *SOX4* or *SOX12* expression levels in studied cell lines (N=3).
